# Supplementary material for: Transforming Cross-Linked Cyclic Dimers of KR-12 into Stable and Potent Antimicrobial Drug Leads
Source: Biomedicines. 2023 Feb 9;11(2):504. doi: 10.3390/biomedicines11020504 (PMC9953701; doi:10.3390/biomedicines11020504)
Supplement: Supplementary file 1 [file biomedicines-11-00504-s001.zip › biomedicines-2182468-supplementary.pdf]

Supporting Information

Transforming cross-linked cyclic dimers of KR-12 into stable and potent antimicrobial drug leads

Taj Muhammad*, Adam A. Strömstedt, Sunithi Gunasekera, Ulf Göransson*

Pharmacognosy, Department of Pharmaceutical Biosciences, Faculty of Pharmacy, Uppsala University, Biomedical Centre, Box 591, SE-75124 Uppsala, Sweden.

***Corresponding authors:**

Taj Muhammad, Pharmacognosy, Department of Pharmaceutical Biosciences, Biomedical Center, Uppsala University, Box 591, SE-75124 Uppsala, Sweden, E-mail: taj.muhammad@farmbio.uu.se

Ulf Goransson, Pharmacognosy, Department of Pharmaceutical Biosciences, Biomedical Center, Uppsala University, Box 591, SE-75124 Uppsala, Sweden, E-mail: ulf.goransson@farmbio.uu.se

Item	Page number
Supplementary Table S1: Net charges, hydropathicity, masses and yield of the cyclic dimers	S2
Supplementary Table S2. MIC of cyclic dimers in MHB and TSB	S3
Supplementary Figure S1: Purity and identity analysis of cross-linked cyclic dimers using RP-HPLC and MS	S4
Supplementary Figure S2: NMR analysis of cross-linked cyclic dimers	S5

Table S1: Sequences, net charge, hydropathicity, and molecular weight of linear peptides

Peptide	Sequence	Net Charge (at neutral pH)[a]	Grand average of hydropathicity[a]	Molecular Weight (Expected)[b]	Molecular Weight (Observed)[b]
cd4-CCPP	CFLRGPGGKRIV CRIKAFLRG PGGKRIVK RIK	+11	- 0.178	3581.49	3582.11
cd4-CC	CFLRGAGGKRIV CRIKAFLRG AGGKRIVK RIK	+11	0.034	3529.41	3529.91
KR-12 (Q5K, D9A)	KRIVKRIKAFLR	+6	- 0.300	1527.97	1528.01
KR-12	KRIVQRIKDFLR	+ 4	- 0.708	1571.93	1572.13
LL-37	LLGDFFRKSKEKI GKEFKRIV QRIKDFLR NLVPRTES	+6	- 0.724	4493.32	4493.98

[a]ExPASy ProtParam tool was used to calculate the molecular weight, net charge, and hydropathy index (http://web.expasy.org/protparam/)

[b]Observed peptides masses presented as $(M+1)^{+}$ have been deconvoluted from $(M+2)^{2+}$, $(M+3)^{3+}$ and $(M+4)^{4+}$ masses.

Table S2: MIC values in two-step microdilution assay, MHB, and TSB

Peptides	MIC (μM)					
	Control[1]		MHB[2]		TSB[3]	
	B. cereus	*B. subtilis*	*B. cereus*	*B. subtilis*	*B. cereus*	*B. subtilis*
LL-37	1.25	1.25	>80	>80	>80	>80
KR-12	2.5	5	>80	>80	>80	>80
KR-12 (Q5K, D9A)	1.25	1.25	>80	>80	> 80	>80
cd4-CCPP	0.312	0.625	>40	>40	>40	>40
cd4-CC	0.625	0.625	>40	>40	>40	>40

[1] The control MICs were determined in a two-step microdilution assay in Tris buffer 10 mM without salts. [2] MHB, Muller-Hinton broth (rich media, unbuffered). [3] TSB, Tryptic Soy broth (rich media, unbuffered).

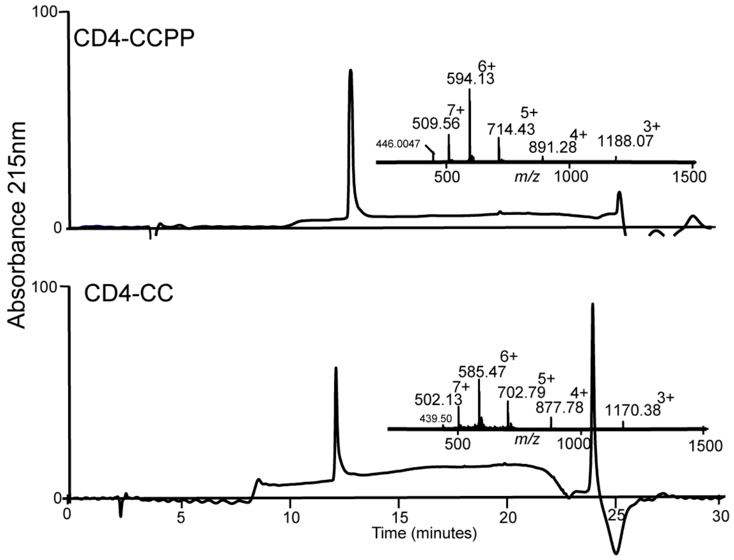

Supplementary Figure S1. Purity and identity analysis of cross-linked cyclic dimers using RP-HPLC and MS. The purity of cross-linked dimers was analyzed at 215 nm and chemical identity was confirmed by LC-MS spectrometer.

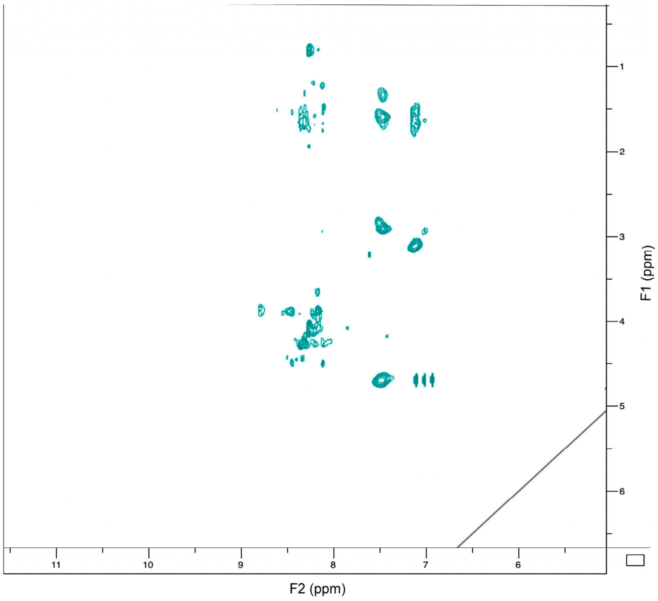

Supplementary Figure S2. NMR Spectroscopy. TOCSY spectrum of cd4-CCPP displayed a broadening of signals and overlapping resonances which made it difficult to assign chemical shift assignments.
